# *Enterobacter aerogenes* and *Enterobacter cloacae*; versatile bacterial pathogens confronting antibiotic treatment

**DOI:** 10.3389/fmicb.2015.00392

**Published:** 2015-05-18

**Authors:** Anne Davin-Regli, Jean-Marie Pagès

**Affiliations:** Transporteurs Membranaires, Chimiorésistance et Drug Design, Facultés de Médecine et Pharmacie, UMR-MD1, IRBA – Aix-Marseille Université, MarseilleFrance

**Keywords:** *Enterobacter aerogenes*, *Enterobacter cloacae*, membrane and transporters, regulation, resistance mechanisms

## Abstract

*Enterobacter aerogenes* and *E. cloacae* have been reported as important opportunistic and multiresistant bacterial pathogens for humans during the last three decades in hospital wards. These Gram-negative bacteria have been largely described during several outbreaks of hospital-acquired infections in Europe and particularly in France. The dissemination of *Enterobacter* sp. is associated with the presence of redundant regulatory cascades that efficiently control the membrane permeability ensuring the bacterial protection and the expression of detoxifying enzymes involved in antibiotic degradation/inactivation. In addition, these bacterial species are able to acquire numerous genetic mobile elements that strongly contribute to antibiotic resistance. Moreover, this particular fitness help them to colonize several environments and hosts and rapidly and efficiently adapt their metabolism and physiology to external conditions and environmental stresses. *Enterobacter* is a versatile bacterium able to promptly respond to the antibiotic treatment in the colonized patient. The balance of the prevalence, *E. aerogenes* versus *E. cloacae*, in the reported hospital infections during the last period, questions about the horizontal transmission of mobile elements containing antibiotic resistance genes, e.g., the efficacy of the exchange of resistance genes *Klebsiella pneumoniae* to *Enterobacter* sp. It is also important to mention the possible role of antibiotic use in the treatment of bacterial infectious diseases in this *E. aerogenes/E. cloacae* evolution.

## Introduction

*Enterobacter* is a genus of a common Gram-negative, facultative anaerobic, rod-shaped, non-spore-forming bacteria belonging to the family *Enterobacteriaceae*. Two of its well- known species, *Enterobacter aerogenes* and *E. cloacae* have taken on clinical significance as opportunistic bacteria and have emerged as nosocomial pathogens from intensive care patients pathogenic, especially to those who are on mechanical ventilation (Mezzatesta et al., 2012).

*Enterobacter aerogenes* was originally named *Aerobacter aerogenes*, and was later included in the genus *Enterobacter* in 1960. In 1971, this species was proposed to be renamed *Klebsiella mobilis* due to its motility conferred by peritrichous flagella and its genetic relatedness to *Klebsiella* genus. It is interesting to note that phenotypic dissimilarities between *E. aerogenes* and the genus *Klebsiella* include not only the motility but also the presence of ornithine decarboxylase (ODC) activity and the lack of urease activity in *E. aerogenes* (Farmer et al., 1985). However, recently, the whole genome sequencing of a multidrug-resistant (MDR) clinical isolate, (including colistin) suggested a possible reclassification of the species in the genus *Klebsiella*, under the name *K. aeromobilis* (Diene et al., 2013). *E. aerogenes* particular phenotype can be attributed to the horizontal acquisition of additional genes from other *Enterobacteriaceae* species and mobile elements that rapidly integrated and translated as easily as its own ancestral heritage (Diene et al., 2013). For example, the flagellar genes and its assembly system have been acquired in bloc from the *Serratia* genus. Plasmid conjugation is a chimera of transposons and genetic elements (conjugation, integration) of various bacterial origins. *E. aerogenes* also contains eight rRNA operons and 87 tRNA associated with the ability to translate imported genes that use different codons, improving its ability to use its integrated foreign genes. *E. aerogenes* has been involved in significant European outbreak between 1993 and 2003 and is considered as the paradigm of opportunistic bacteria.

Species of the *E. cloacae* complex are widely encountered in nature, but they are also pathogens: *E. cloacae* and *E. hormaechei* are most frequently isolated from human clinical specimens. Thus, *E. cloacae* is among the most common *Enterobacter* sp. causing only nosocomial infections in the last decade and a lot has been published on the antibiotic-resistance features of these microorganisms. Despite the relevance of *E. cloacae* as a nosocomial pathogen, the pathogenic mechanisms and factors contributing in the disease associated with the *E. cloacae* complex are not understood yet; this could be due to the scarcity and the dispersion of information available. Its ability to form biofilms and to secrete various cytotoxins (enterotoxins, hemolysins, pore-forming toxins) are important for its pathogenicity (Mezzatesta et al., 2012). Some genotypes and species, have previously exhibited some associations with clinical specimens, in particular urines and sputum, when clonal outbreaks with members of the *E. cloacae* complex were rare (Izdebski et al., 2014). Interestingly, due to the diffusion of most frequent extended spectrum β-lactamases (ESBL) and carbapenemases in this species, *E. cloacae* has now become the third broad spectrum *Enterobacteriaceae* species involved in nosocomial infections after *Escherichia coli* and *K. pneumoniae* (Potron et al., 2013; Jarlier and INVS, 2014).

## Epidemiology and Infections

**FIGURE 1:**
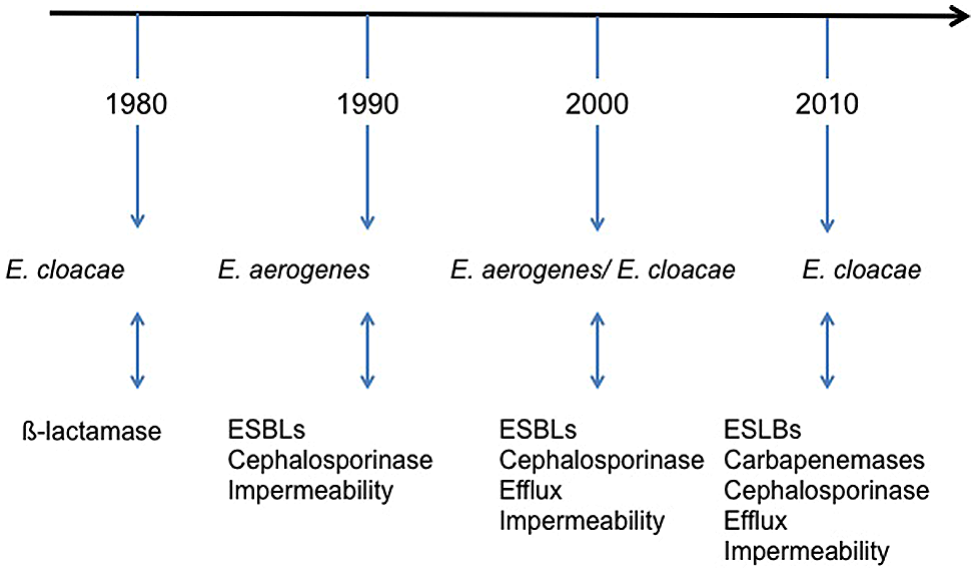
**Schematic illustration of the timing of *Enterobacter* species during the emergence of resistance outbreaks in France hospitals (Arpin et al., 1996; Bornet et al., 2000; Chevalier et al., 2008; Lavigne et al., 2012; Mezzatesta et al., 2012; Anastay et al., 2013; Robert et al., 2014)**. ESBLs, extended-spectrum β-lactamases.

*Enterobacter aerogenes* is isolated as human clinical specimens from respiratory, urinary, blood, or gastrointestinal tract (Langley et al., 2001). Epidemiology of this species has been particular in Europe: it has regularly been involved in nosocomial infections outbreaks since 1993, particularly in the Western Europe (Georghiou et al., 1995; Grattard et al., 1995; Allerberger et al., 1996; Arpin et al., 1996; Davin-Regli et al., 1996; De Gheldre et al., 1997; Jalaluddin et al., 1998). Until, 2003, *E. aerogenes* was considered as an important emerging MDR pathogen, particularly in ICUs (Bosi et al., 1999; Chevalier et al., 2008; **Figure 1**). The situation in 1990s in Europe pointed to the dispersion of an epidemic clone and, since then, it has been extensively detected in European hospitals and health care facilities. The event fitted in with the international spread of the ESBL TEM-24 (*bla*_TEM-24_) harbored by an epidemic plasmid (Bosi et al., 1999). The prevalence of *Enterobacter* sp. infections in clinical wards has also increased due to the introduction of extended-spectrum cephalosporins and carbapenems in the antibiotic therapy (Arpin et al., 1996; Anastay et al., 2013). The consequence of this antibiotherapy is the emergence of “pan-drug *E. aerogenes* isolates” resistant to last-line antibiotics such as carbapenems and also to colistin, for which no therapeutic option was available (Chevalier et al., 1999; Thiolas et al., 2005; Diene et al., 2013). Interestingly, the role of efflux mechanism in *E. aerogenes* resistance has been studied within an 8 years of period. This study indicated a noticeable increase of the prevalence of an efflux mechanism, susceptible to pump inhibitor, in clinical isolates collected during this period (Chevalier et al., 2008). After the emergence of ESBL in *E. aerogenes* and the characterisation of porin mutations in clinical isolates, this role of efflux mechanism highlights a new step in the adaptative evolution in *E. aerogenes* (Charrel et al., 1996; Malléa et al., 1998; Gayet et al., 2003).

**FIGURE 2:**
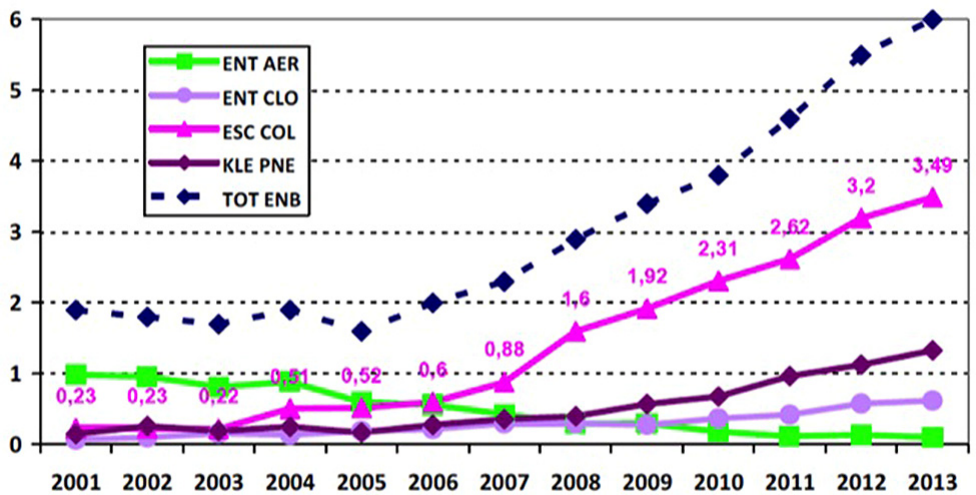
**Distribution of the main species of *Enterobacteriaceae*-ESBL (*n* for 10,000 patient days): evolution 2002–2013 from the French national coordination of MDRB surveillance (Carbonne et al., 2013; Jarlier and INVS, 2014)**. ENT AER, *Enterobacter aerogenes*; ENT CLO, *E. cloacae*; ESC COL, *E. coli*; KLE PNE, *Klebsiella pneumoniae*; TOT ENB, Total *Enterobacteriaceae*; ESBLs, extended-spectrum β-lactamases.

Since 2010, *E. aerogenes* in France is the fifth highest *Enterobacteriaceae* and the seventh highest Gram-negative *Bacillus* responsible for notorious nosocomial infections (Carbonne et al., 2013; **Figure 2**). Despite its intrinsic resistance to ampicillin and constant expression of ESBL that is associated with other resistance mechanisms contributing to MDR phenotype, its prevalence has significantly dropped (reduction factor of 20) in France (Anastay et al., 2013; Jarlier and INVS, 2014). Its position was displaced in the context of hospital acquired infections, because of the dramatic rise of the *E. coli* pandemic clone O25:H4-ST131 along with *K. pneumoniae* and *E. cloacae*, ESBL, and/or carbapenemase producing strains. Although, *E. aerogenes* causes septic shock more readily in patients thus leading to a higher mortality rate (Song et al., 2010; Lavigne et al., 2012), *E. cloacae* is now the most frequently observed clinical isolate among *Enterobacter* sp. It can be associated with the dissemination of actual epidemic plasmids bearing most prevalent resistant genes and expressing new β-lactamases or carbapen**e**mases (**Figure 2**).

*Enterobacter cloacae* is ubiquitous in terrestrial and aquatic environments (water, sewage, soil, and food). The species occurs as commensal microflora in the intestinal tracts of humans and animals and is also pathogens in plants and insects. This diversity of habitats is mirrored by the genetic variety of *E. cloacae* (Mezzatesta et al., 2012). Recently, MLST and PFGE epidemiological methods data revealed world circulation of several epidemic clonal complexes (Izdebski et al., 2014).

It is also a well-known nosocomial pathogen contributing to bacteremia, endocarditis, septic arthritis, osteomyelitis, and skin/soft tissue infections, and lower respiratory tract- urinary tract and intra-abdominal infections (Fata et al., 1996). *E. cloacae* tends to contaminate various medical, intravenous, and other hospital devices (Dugleux et al., 1991). Nosocomial outbreaks have also been associated with the colonization of certain surgical equipment and operative cleaning solutions (Wang et al., 2000). Since a decade, *E. cloacae* has been repeatedly reported as a nosocomial pathogen in neonatal units and several outbreaks of infection have been reported (Fernandez-Baca et al., 2001; Pestourie et al., 2014). Today, variability among strains are less frequent and outbreaks due to clonal *E. cloacae* hyper-producing AmpC β-lactamase and ESBL carrier isolates are described from neonate specimens, adults urines/feces samples or from environmental samples (Pestourie et al., 2014).

*Enterobacter cloacae* has an intrinsic resistance to ampicillin, amoxicillin, first-generation cephalosporins, and cefoxitin owing to the production of constitutive AmpC β-lactamase. It exhibits a high frequency of enzymatic resistance to broad-spectrum cephalosporins. Resistance of *Enterobacter* sp. to third-generation cephalosporins is most typically caused by overproduction of AmpC β-lactamases, and thus treatment with third-generation cephalosporins may select for AmpC-overproducing mutants. AmpC overproduction is due to the derepression of a chromosomal gene or by the acquisition of a transferable *ampC* gene from plasmids or other mobile elements. The AmpC plasmid-mediated resistance is distinguished from chromosomal enzyme production because they are not inducible. However they represent a problem due to its increasing prevalence among clinical isolates. The enzyme confers a resistance to third-generation cephalosporins and ureido- and carboxy-penicillins and is not inhibited by common inhibitors of β-lactamases. Fourth-generation cephalosporins retain reasonable activity against derepressed strains, but if strains are also ESBL producers, they become resistant to this antibiotic class. The prevalence of ESBL and CTX-M producers represented approximatively 5% of the isolates in the recent studies and ESBLs are most often plasmid-mediated. These characteristics, associated with the frequent endogenous intestinal carriage of *E. cloacae*, may result in abnormally high levels in the bowels of hospitalized patients, especially those who have received cephalosporins (Potron et al., 2013).

## Enzymatic Barrier and Antibiotic Resistance

The production of β-lactamases is the prominent mechanism responsible for β-lactam resistance in most of these species. *E. aerogenes* strains have a broad ability to develop antibiotics resistance mechanisms (Miro et al., 1995). They naturally express a chromosomal AmpC β-lactamase type cephalosporinase at low level (group 1 Bush) that induces resistance to first-generation cephalosporins (Freney et al., 1988). Chromosomal acquired β-lactams resistance mechanisms induce the overproduction of chromosomal AmpC cephalosporinase: this results from an induction during a third-generation cephalosporin treatment or by a mutation in the AmpR repressor, and generates a resistance to almost all β-lactams (Preston et al., 2000). Moreover, it has been described that *E. aerogenes* strains harboring cephalosporinase AmpC gene, integrated the gene of chromosomal origin (*bla*CMY-10) on a large plasmid (130 kb), contributing to a systematic gene transmission even in the absence of antibiotic pressure (Lee et al., 2003).

In 1993 appeared the first cases of nosocomial infections caused by strains with resistance to common β-lactam antibiotics due to ESBL (Pitout et al., 1998). The ESBL TEM- 24 associated to *E. aerogenes* clonal dissemination in France was constantly reported (Neuwirth et al., 1996; Bosi et al., 1999; Bertrand et al., 2003). Other ESBLs of TEM type or CTX-M type (ex CTX-M-2) are often identified but TEM-24 remains associated with preferential conjugative plasmid of this species (Arpin et al., 2002; Dumarche et al., 2002; Biendo et al., 2008; Kanamori et al., 2012). Due to the well-described modification of porins expression and recent dissemination of plasmid bearing carbapenemases, a number of imipenem-resistant clinical strains have come up (Miro et al., 1995; Bornet et al., 2000; Biendo et al., 2008; Lavigne et al., 2012). Carbapenemases of NDM and VIM types are now, as anticipated, reported in *E. aerogenes* in India and those for the serine protease group as KPC or class D β-lactamases possessing carbapenemase properties as OXA-48 types are described in Europe/Asia (Khajuria et al., 2014; Torres et al., 2014).

Similarly to *E. aerogenes*, *E. cloacae* is also naturally resistant to ampicillin, amoxicillin–clavulanic acid, cephalothin, and cefoxitin by low production of the natural inducible cephalosporinase of Bush group 1 (class C). They are capable of overproducing AmpC β-lactamases by blocking the repression of a chromosomal gene or by the acquisition of a transferable *ampC* gene on plasmids conferring the resistance to third-generation cephalosporins (Nauciel et al., 1985; Zaher and Cimolai, 1997). Cefepime alone can keep its activity (Sanders and Sanders, 1997). Clinical AmpC resistance represents 50% of the isolates and frequently co- exists with the expression of ESBL. In 1989, appeared the first nosocomial isolate cases bearing plasmidic ESBL causing also resistance to third generation cephalosporins except cefamycins (De Champs et al., 1989). Together, these enzymes are responsible for a global resistance to all β-lactams except carbapenems (Pitout et al., 1997). In the last decade, *E. cloacae* has emerged as the third most common *Enterobacteriaceae* resistant to third generation cephalosporins with enteric *E. coli* and *K. pneumoniae* (Jarlier and INVS, 2014). Imipenem remains the most effective molecule for treating *E. cloacae* infections. Since then, various ESBL of TEM, SHV, and CTX -M types have been characterized in *E. cloacae* including resistant TEM inhibitors or IRT (for inhibitor-resistant TEM**;** Arpin et al., 2002; Szabo et al., 2005; Galas et al., 2008). However, among ESBL producers, some sub-clones are now identified, associated with CTX-M-3 and 15 production, when other TEM or SHV (SHV-12 for example) types are also associated with epidemic-episodes-involved isolates. Diffusion of *E. cloacae* producing CTX-M-15 ESBL is the consequence of the wide dissemination of identical or related plasmids harboring the CTX-M-15 gene firstly identified in the epidemic *E. coli* clone, and the CTX-M β-lactamases are now the most prevalent ESBL globally (Hammami et al., 2012).

In recent years, clinical isolates resistant by producing carbapenemases have been identified (Nordmann et al., 1993; Galani et al., 2005). In 2010, CDC first reported carriage of NDM-1 in *E. cloacae* from patients who received medical care in India. Especially in Asia, strains harboring metallo-β-lactamases as IMP -type enzymes, NDM, GIM, VIM, and serine carbapenemase type KPC have been described (Huang et al., 2012; Dai et al., 2013; Hamprecht et al., 2013; Jaskulski et al., 2013). The OXA-48 type serine carbapenemase is the most prevalent because its gene is located on a plasmid, associated to the *bla-CTX-M-15* gene coding ESBL, thus explaining its spread and the associated resistance (Potron et al., 2013; Torres et al., 2014). A hike in the imipenem resistance rate in *E. cloacae*, from 0.4 to 8 %, has been observed (Lee et al., 2005; Poirel et al., 2007; Robert et al., 2014). An epidemic study concerning *E. cloacae* blood stream infections indicated a 25% production of metallo-β-lactamase in corresponding strains (Khajuria et al., 2014). Thus, the decreased susceptibility to carbapenems in hospital acquired *E. cloacae* isolates might arise via stepwise accumulations of MDR determinants in different clones. Today, *E. cloacae* is the second *Enterobacteriaceae* carrying carbapenemase and strains co-expressing two carbapenemases has been reported (Izdebski et al., 2014).

Regarding the aminoglycosides, the major mechanism of resistance of the *Enterobacteriaceae* is due to aminoglycoside-modifying enzymes that are often plasmid-encoded, but it may also be associated with transposable elements. These enzymes are assigned to three groups: acetyltransferases (acetylation of an amino group/AAC), phosphotransferases (phosphorylation of a hydroxyl group/APH), and adenylyltransferases (adenylylation of a hydroxyl group/AAD or ANT). Plasmid exchanges and disseminations of transposons facilitate the rapid acquisition of resistance phenotypes (Mezzatesta et al., 2012).

The resistant strain varies from 0 to 51% resistance for gentamicin, and 0 to 34% for amikacin (Sanders and Sanders, 1997). In 2013, an important epidemiological study confirmed that the aminoglycoside-modifying genes involved in aminoglycoside-clinical resistance were *aac*(*3*)*-IIa*, *aac*(*6*′)*-Ib*, *and ant*(*2*′′)*-Ia*, genes that confer resistance to tobramycin, gentamicin, and amikacin (Miró et al., 2013). Strains have frequently more than one enzyme (Miró et al., 2013). This enzymatic type resistance is associated in 77% of clinical isolates in China to other plasmid genes (*armA*, *rmtB*; Huang et al., 2012). Among these, the aminoglycoside AAC(6′)-Ib is the most common cause of amikacin resistance among members of the *Enterobacteriaceae* family. In a previous study, it was observed that over 40% of the *E. cloacae* isolates had the *aac*(6′)-Ib gene, although many of the isolates with this gene were susceptible to amikacin and gentamicin, which were the most active of all tested drugs (Kim et al., 2009).

The enzymatic resistance to fluoroquinolones has been recently described and attributed to a two-point mutation allele of *aac*(*6*′)-*Ib* [named *aac*(*6*′)-*Ib*-*cr*], the aminoglycosides resistance enzymatic determinant, which acetylates ciprofloxacin and norfloxacin (Huang et al., 2012). A systematic molecular survey reporting prevalence and characteristics of *aac*(*6*′)*-Ib-cr* in Korea, characterized a high prevalence of the mechanism (23%) in *E. cloacae* (Huang et al., 2012). Dissemination of this new enzymatic resistance mechanism occurs since the *aac*(6′)*-Ib-cr* is highly associated with *bla*_OXA-1_, IS*CR1*, and class 1 integron. This supports the previous finding where *aac*(*6*′)*-Ib-cr* was located upstream of *bla*_OXA-30_ (synonymously called *bla*_OXA-1_) in complex class 1 integron, In37 containing IS*CR1* (Quiroga et al., 2007). A genetic linkage between *aac*(*6*′)*-Ib-cr* and *bla*_CTX-M-15_ has been demonstrated (Huang et al., 2012).

## Membrane Barrier and Antibiotic Resistance

### Porin and Membrane Permeability

Carbapenems are the most powerful agents for the treatment of serious nosocomial infections caused by MDR *Enterobacteriaceae*. Due to the imipenem use, it was rapidly reported a decreased penetration of β-lactams due to a change in the expression of porins in *E. aerogenes* isolates. Charrel et al. (1996) showed that MDR strains of *E. aerogenes* exhibited a characteristic phenotype associated with an altered expression of porins and then successive studies comforted description of more frequent MDR strains in treated patients by β-lactams (Bornet et al., 2000; Fernandez-Cuenca et al., 2006). This mechanism of resistance is reversible upon discontinuation of treatment (Bornet et al., 2000) and progressive during treatment. Among intermediate strains which are susceptible to imipenem but resistant to ertapenem, there is a loss of porin Omp35 but the expression of porin Omp36 is preserved. When treatment with imipenem continues, the disappearance of two porins and resistance to all carbapenems is noted (Lavigne et al., 2013). Recently, a novel mechanism of resistance has been observed in a clinical strain where the antibiotic cannot be efficiently translocated through a mutated porin (see Mutation and Antibiotic Resistance). Additionally, imipenem and carbapenemase KPC type have been described as responsible for resistance to carbapenems associated to decrease in membrane permeability (Jaskulski et al., 2013). The conductance and selectivity of these porins, Omp35, and Omp36, correspond to the properties obtained with OmpC and OmpF of *E. coli* (Bornet et al., 2004; James et al., 2009). Moreover, several studies have further described a decrease in production of these porins in resistant isolates (Bornet et al., 2000; Yigit et al., 2002; Gayet et al., 2003; Doumith et al., 2009; Tran et al., 2009). Two major outer membrane porins have been identified in *E. cloacae* and studied by liposome swelling assays (Lee et al., 1992). These porins are involved in the carbapenem susceptibility (Raimondi et al., 1991; Lee et al., 1992) and exhibit important cross antigenicity with the *E. aerogenes* porins in specific key parts, e.g., eyelet region, membrane insertions, subunit connections (Malléa et al., 1995). In addition, their respective involvement in β-lactam and fluoroquinolone uptake has been reported (Chevalier et al., 2000; James et al., 2009).

Interestingly, the expression of porin in *Enterobacteriaceae* is rapidly and notably altered by various stress compounds present in the external medium (Dupont et al., 2007). During the first hours of incubation in the presence of salicylate, novobiocin, norfloxacin a significant increase of OmpX is observed and this overexpression negatively controls the synthesis of porins (Dupont et al., 2007).

### Efflux and Membrane Permeability

Furthermore, an efflux mechanism that is involved in the expelling of molecules from the bacteria such as fluoroquinolones, tetracycline, and chloramphenicol is active in *Enterobacter* sp. (Malléa et al., 1998). This mechanism is highly efficient since the AcrAB–TolC efflux pump can eject about 80–90% of the norfloxacin during the first 10–15 min (Malléa et al., 1998). Interestingly, this process is energy-dependent and requires the membrane energy (proton motive force) as extensively described (for a recent review see Nikaido and Pagès, 2012). Approximately 40% of MDR clinical strains have an active efflux (Chevalier et al., 2008). The EefABC and AcrAB–TolC efflux genes of *E. aerogenes* have been described and their involvement in antibiotic exportation has been studied (Pradel and Pagès, 2002; Masi et al., 2005, 2006; Martins et al., 2010). Several studies on *E. cloacae* have also reported the presence of efflux pumps belonging to RND and MATE families (Pérez et al., 2007; He et al., 2011). In addition, the AcrAB–TolC and OqxAB genes have been characterized in *E. cloacae* clinical resistant isolates (Pérez et al., 2007, 2012; Veleba et al., 2013). In *E. aerogenes* and *E. cloacae*, the sequence similarities and biological activity are particularly high in AcrAB–TolC (Pradel and Pagès, 2002; Pérez et al., 2007). Moreover, various chemicals such as salicylate, chloramphenicol, and imipenem are also able to trigger the genetic cascade controlling the expression of *Enterobacter* AcrAB–TolC pump (Davin-Regli et al., 2008). The regulation seems to be associated with the internal concentration of chemicals that plays a key role during the switch on of the cascade that provides the efflux expression (Valade et al., 2013).

MarA acts as a key regulator for the expression of porin genes and *tolC* in *Enterobacteriaceae* (Levy, 2002; Piddock, 2006; Alekshun and Levy, 2007; Davin-Regli et al., 2008). SoxS is another key transcriptional regulator that is positively controlled by oxidative stress and can trigger the MarA expression (Masi and Pagès, 2013). Some *Enterobacteriaceae* sp., such as *Enterobacter, Klebsiella, Salmonella*, have an additional global regulator, RamA. It plays a strategic role in controlling both the porins and the efflux expression, either directly or *via* the MarA cascade. This coordinated control of influx and efflux directly and efficiently governs the intracellular accumulation of antibacterial agents. Importantly, this internal accumulation of antibacterial molecules below the threshold corresponding to the MIC can favor the emergence and acquisition of additional mechanisms of resistance such as target mutation, production of detoxifying enzymes (e.g., β-lactamases, acetyltransferase, etc), and contributing to the extension of MDR phenotype (Nikaido and Pagès, 2012; Masi and Pagès, 2013).

Regarding the active structure of efflux pumps involved in *E. aerogenes* and *E. cloacae*; we can hypothesize that a common structural organization is conserved, due to the high conserved homology between *Enterobacter* and *E. coli*. This structural organization can be similar to the recent description of the AcrAB–TolC complex in *E. coli* (Du et al., 2014).

## Mutations and Antibiotic Resistance

Regarding the β-lactam antibiotics, the resistance due to target mutation occurs incidentally in *Enterobacter* sp. However, the diverse β-lactamases identified today is the result of a series of mutations that have successively appeared in the original β-lactamases TEM-1/2, SHV-1, OXA-1.

Furthermore, strains in which AmpC cephalosporinase was derepressed have been affected by mutations affecting AmpR-promoter recognition site. Mutations that are best known and studied are those that affect the target of fluoroquinolones and more recently those responsible for polymyxin resistance. As a matter of fact, the quinolones were widely prescribed antimicrobial agents because of their proven safety, high oral bioavailability, multiple approved indications, and bactericidal activity. Consequently, in the microbial population, a variety of amino acid alterations arose from mutations within quinolone resistance-determining regions (QRDRs) of cellular target genes *gyrA* and *parC* and conferred high-level resistance. This is one of the most common resistance mechanisms identified among clinical isolates of *Enterobacter*, despite recent characterization of plasmid-mediated quinolone resistance (PMQR) genes (*qnrA*, *qnrS*, *aac*(*6*′)*-Ib-cr*, *qepA*, and *oqxAB*; Park et al., 2009; Kanamori et al., 2012). In *E. cloacae* the plasmid-borne QnrA and QnrS resistances inducing protection from the DNA binding of fluoroquinolones are observed, but such mechanisms confers low-level resistance when present alone (Corkill et al., 2005; Poirel et al., 2005; Huang et al., 2012; Kanamori et al., 2012). However, such PMRQ mechanisms have got an efficient dissemination and are found in over 60% of the strains, because were found to be co-carried with various ESBLs or AmpC-type β-lactamases on the same plasmid (Park et al., 2009; Huang et al., 2012). Finally, associated to active efflux, target mutations are the most efficient resistance mechanisms resulting in high MICs values, while PMRQ mechanisms confer only an additive effect on the level of fluoroquinolones resistance.

Finally, pan-drug resistance is not an exceptional phenotype in *E. aerogenes*, since resistant strains to all antibiotics, including colistin *pmrA* substitution, were isolated and described to be associated with colistin resistance (Thiolas et al., 2005; Diene et al., 2013).

Regarding the permeation pathway, it is important to mention that during the last decade, we observed the emergence of well-located mutation inside the pore constriction of the Omp36 (OmpC like porin of *E. aerogenes*) that generate a strong resistance against β-lactams (Dé et al., 2001; Thiolas et al., 2004). This specific mutation altering the pore characteristics impairs the diffusion of all β-lactams including cephalosporins and carbapenems, represents the first type of an adaptative mutation of bacterial porin in a resistant clinical isolates of *Enterobacteriacea****e*** (Chevalier et al., 1999; Thiolas et al., 2004). Interestingly, the intensity of MIC modification conferred by the specific residues depends on the structure and charge of the antibiotic molecules. A recent study reports the molecular simulations and dynamics of β-lactams inside the wild type and mutated channel during the travel of the molecule from outside to the periplasmic space (Vidal et al., 2005; James et al., 2009; Hajjar et al., 2010a,b). These data illustrates the adaptive pressure that has governed the selection and the preservation of these specific residues that filter the diffusion of charged solutes. The amino acids involved represent the first defense against the penetration of harmful compounds and support the pioneer investigations reporting the difference in β-lactam susceptibility depending on porin (Pagès et al., 2008).

Recently, in the context of IMI-Translocation consortium (www.translocation.eu), the genomes of various clinical isolates have been sequenced and the preliminary analyses have reported several mutations in resistant strains that are located in the regulators and membrane proteins (data not shown).

## Regulation of Membrane-Associated Mechanisms of Resistance

Various studies on antibiotic resistance in *E. aerogenes* and *E. cloacae* have enlightened on a group of AraC family regulators including MarA, RamA, SoxS, and RobA, which are associated with a phenotype of low-level susceptibility to several antibiotics and biocides by inducing the overexpression of the efflux pump (for a review see Davin-Regli et al., 2008; Davin-Regli and Pagès, 2012; Pérez et al., 2012). Interestingly, the role of *marA* and *ramA* has been described also in the downregulation of porins and the subsequent resistance to β-lactams in *E. aerogenes* that completes the MDR phenotype of clinical resistant strains (Chollet et al., 2002, 2004). Recent work has shown that the expression of another AraC- regulator, *rarA*, contribute to a multidrug-resistance phenotype, generated *via* the activation of efflux (Veleba et al., 2013). This regulator also has a role in the development of tigecycline resistance (Veleba et al., 2013). Thus, the regulation of MDR in *Enterobacter* is quite complex and redundant (Davin-Regli et al., 2008; Lawler et al., 2013) and contributes to the rapid adaptation of the clinical isolate *via* the porin and efflux balance (Bornet et al., 2000, 2004). Moreover, it has been demonstrated that some two component system (TCS) regulators such as OmpR-EnvZ also play a key role in the control of porin expression in addition to OmpX and the small RNAi or proteins as H-NS that govern the OmpF/OmpC balance in *E. coli* or efflux pump elements synthesis in *E. aerogenes*, respectively (Stoorvogel et al., 1991; Masi et al., 2005; Dupont et al., 2007).

Interestingly, regarding the genetic control of the pump expression; activators MarA, RamA, and RarA, and repressors MarR, RamR, and AcrR, could be intimately associated at a global and local level to conjointly organize the resistance in clinical *Enterobacter* isolates (Davin-Regli et al., 2008). At this moment, it is also important to mention that the RamA regulator is described in *Enterobacter*, *Salmonella*, *and Klebsiella*, but not reported in *Escherichia* in contrast to the Mar regulon (Lawler et al., 2013).

**FIGURE 3:**
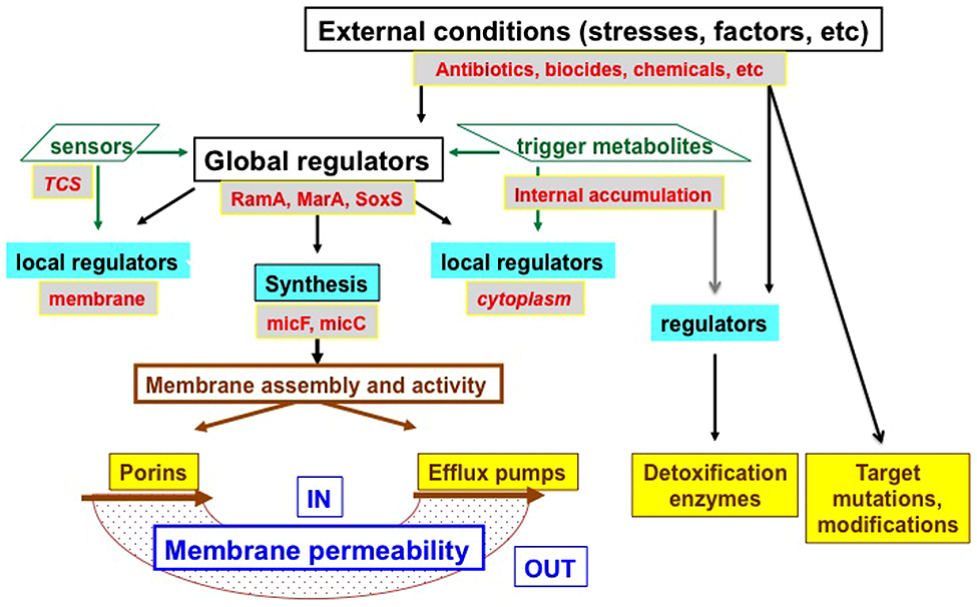
**Schematic illustration of resistance mechanism and their regulations**. Different levels of regulation are presented: (i) Transcriptional and translational levels controlled by various sensors [two components systems (TCS), such as EnvZ/OmpR for porins], global regulators (RamA, MarA, SoxS are indicated here), and local regulators (AcrR for efflux pumps and OmpX for porins), the accumulation of trigger metabolites inside the bacterial cell can also trigger the expression via local or other regulators, MicF and MicC represent the small interfering RNA controlling porin mRNA stability. (ii) Translational and final membrane assembly in a functional conformation (via chaperones and membrane factors. Porins represent Omp35, Omp36; Efflux pumps represent the AcrAB–TolC family. IN, bacterial cytoplasm; OUT, external medium.

An illustration of the sophisticated regulation of the various resistance mechanisms in *Enterobacter* is presented in **Figure 3**.

## Conclusion

During the last decade, we observed the rise and the fall of several infectious episodes due to resistant *Escherichia*, *Enterobacter*, *Klebsiella* strains in French and European hospitals. Regarding *Enterobacter*, we can note the successive waves of *E. cloacae*, followed by *E. aerogenes* and now again *E. cloacae* reported in hospital wards (Potron et al., 2013). This bacterial species is a member of the ESKAPE group recently described as the main contributor to the health human infection problem (Boucher et al., 2009; Rice, 2010). Consequently, it is important to examine the various ways used by *E. aerogenes* and *E. cloacae* to detect and respond to the modification of environmental conditions and the presence of drugs in the medium.

Interestingly, the two *Enterobacter* species, *aerogenes* and *cloacae*, present highly preserved regulation mechanisms acting to modulate the expression of porins integrated into the outer membrane: for example OmpX the small outer membrane protein, plays a role in controlling the production of the OmpF-like porin (Omp35) and OmpX overproduction is reported in clinical isolates showing a porin failure (Stoorvogel et al., 1991; Dupont et al., 2007). This control of an outer membrane protein (OmpX) on the synthesis of the outer membrane porin, in addition to the major regulator of the resistance cascade that are fully active, are present in various clinical isolates. This suggests a common evolution path and the selection of a common regulation cascade involved in the membrane adaptation to environmental stresses (Gayet et al., 2003). About the drug transporters, it is clear that AcrAB–TolC system, OqxAB, EmrE, MdfA, and MacA are present in the two species (see data bank for a complete description). In addition, regarding Mar, Ram, and Sox regulators all of them are preserved and active in the triggering of antibiotic resistance. Interestingly, the redundant global regulatory control, Mar and Ram, are reported in *E. aerogenes* and *E. cloacae* (Veleba et al., 2013). The close species proximity is reinforced by the presence of similar regulators and adaptive response and support the description of these species in the human infection and their response face to antibiotic therapy.

## Conflict of Interest Statement

The authors declare that the research was conducted in the absence of any commercial or financial relationships that could be construed as a potential conflict of interest.
